# Controlling the phase locking of stochastic magnetic bits for ultra-low power computation

**DOI:** 10.1038/srep30535

**Published:** 2016-07-26

**Authors:** Alice Mizrahi, Nicolas Locatelli, Romain Lebrun, Vincent Cros, Akio Fukushima, Hitoshi Kubota, Shinji Yuasa, Damien Querlioz, Julie Grollier

**Affiliations:** 1 Unité Mixte de Physique CNRS, Thales, Univ. Paris-Sud, Université Paris-Saclay, 91767 Palaiseau, France; 2 Centre de Nanosciences et de Nanotechnologies, CNRS, Univ Paris-Sud, Université Paris-Saclay, 91405 Orsay France; 3 Spintronics Research Center, National Institute of Advanced Industrial Science and Technology (AIST), Tsukuba, Japan

## Abstract

When fabricating magnetic memories, one of the main challenges is to maintain the bit stability while downscaling. Indeed, for magnetic volumes of a few thousand nm^3^, the energy barrier between magnetic configurations becomes comparable to the thermal energy at room temperature. Then, switches of the magnetization spontaneously occur. These volatile, superparamagnetic nanomagnets are generally considered useless. But what if we could use them as low power computational building blocks? Remarkably, they can oscillate without the need of any external dc drive, and despite their stochastic nature, they can beat in unison with an external periodic signal. Here we show that the phase locking of superparamagnetic tunnel junctions can be induced and suppressed by electrical noise injection. We develop a comprehensive model giving the conditions for synchronization, and predict that it can be achieved with a total energy cost lower than 10^−13^ J. Our results open the path to ultra-low power computation based on the controlled synchronization of oscillators.

Superparamagnetic tunnel junctions present a number of advantages for computation. First, they can be downscaled to atomic dimensions^1^. Secondly, because the energy barrier separating the two magnetic configurations is small, low current densities can lead to significant action of spin torques on magnetization switching^2^. But how can they be harnessed for applications? A first option is to use superparamagnetic tunnel junctions as sensors. Indeed, thanks to their high sensitivity to electrical currents they are able to detect weak oscillating signals^3^,^4^,^5^ through the effect of stochastic resonance^6^. A second option is to use them as building blocks of computing systems leveraging the synchronization of oscillators for processing^7^,^8^. It has been recently recognized that coupled nano-oscillators are promising brain-inspired systems for performing cognitive tasks such as pattern recognition^9^,^10^,^11^,^12^,^13^,^14^,^15^,^16^. Like neurons in some parts of the brain, they compute by synchronizing and desynchronizing depending on sensory inputs^17^. However, such systems require a high number of oscillators, that each need to be powered. Using superparamagnetic tunnel junctions would allow orders of magnitude gain in power consumption. In addition, by shrinking their dimensions they can be fabricated from the same magnetic stack as stable junctions, allowing for densely interweaving oscillations and memory.

Nevertheless there are a number of prerequisites to be able to use superparamagnetic tunnel junctions for computational purposes. In particular, it is necessary to identify handles providing control over their synchronization and to model accurately the associated physics for simulating large scale systems of interacting oscillators. Here we show experimentally that we can induce the phase-locking of a superparamagnetic tunnel junction to a weak periodic signal through the addition of a small electrical noise, and that we can suppress the phase-locking by adding more noise. While the stochastic behavior of most systems becomes unpredictable when shrunk to nanometer scale, the dedicated model we develop here encompasses all our experimental results. The quantitative agreement between model and experiments allows predicting the power consumption of computing systems harnessing phase-locking of superparamagnetic tunnel junctions.

## Experimental Results

**Figure 1:**
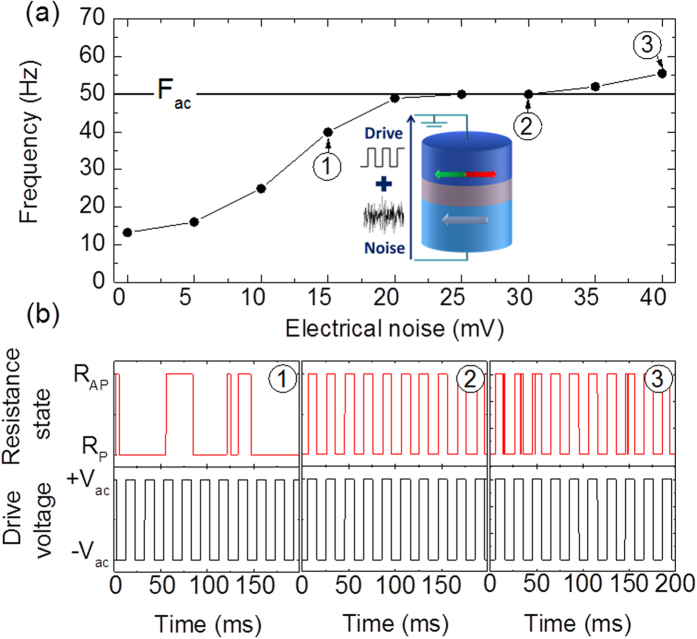
Controlling the phase locking of a superparamagnetic tunnel junction through electrical noise: experimental results. A square periodic voltage of amplitude V_ac_ = 63 mV and frequency F_ac_ = 50 Hz as well as white Gaussian electrical noise are applied to the junction. (**a**) Inset: schematic of the superparamagnetic tunnel junction driven by a periodic square voltage and electrical noise. Main: junction’s mean frequency as a function of electrical noise amplitude (standard deviation σ_Noise_). (**b**) Times traces of the junction’s resistance (top) and applied voltage (bottom) for three different levels of noise with standard deviations: (1) σ_Noise_ = 15 mV, (2) σ_Noise_ = 30 mV and (3) σ_Noise_ = 40 mV.

We study experimentally superparamagnetic tunnel junctions with an MgO barrier and a CoFeB free layer of dimensions 60 × 120 × 1.7 nm^3^ (details in Methods). As depicted in the inset of Fig. 1a, we evaluate their ability to phase lock to a weak square periodic drive voltage in the presence of electrical white noise, at room temperature. We set the drive frequency at *F*_*ac*_ = 50 Hz and the drive amplitude at *V*_*ac*_ = 63 mV, which corresponds to approximately 25% of the voltage threshold for deterministic magnetization switching at 0 K. Figure 1a shows how the mean frequency of the stochastic oscillator evolves when the amplitude of the electrical noise is increased.

We observe three different regimes, illustrated in Fig. 1b. As can be seen in the first panel, the jumps in the junction resistance, corresponding to reversals of the magnetization, remain stochastic for small values of injected electrical noise. In addition, the junction mean frequency is lower than the drive frequency. Usually, adding noise to a system tends to destroy its coherence and is detrimental to the occurrence of a synchronized regime. On the contrary, in our case, by increasing the electrical noise amplitude, we can increase the junction’s mean frequency towards the drive frequency. Eventually, for an optimal range of electrical noise (between 20 and 30 mV), we observe both frequency locking (as evidenced from the plateau in Fig. 1a), and phase locking to the driving signal (as shown in panel 2). In this second regime, electrical noise optimally assists the periodic drive to overcome the voltage threshold for magnetization switching at every oscillation of the drive voltage^18^,^19^,^20^. In the third regime (panel 3), higher amplitude electrical noise induces unwanted switches of the magnetization and prevents synchronization.

### Analytical model and simulations of phase-locking

**Figure 2:**
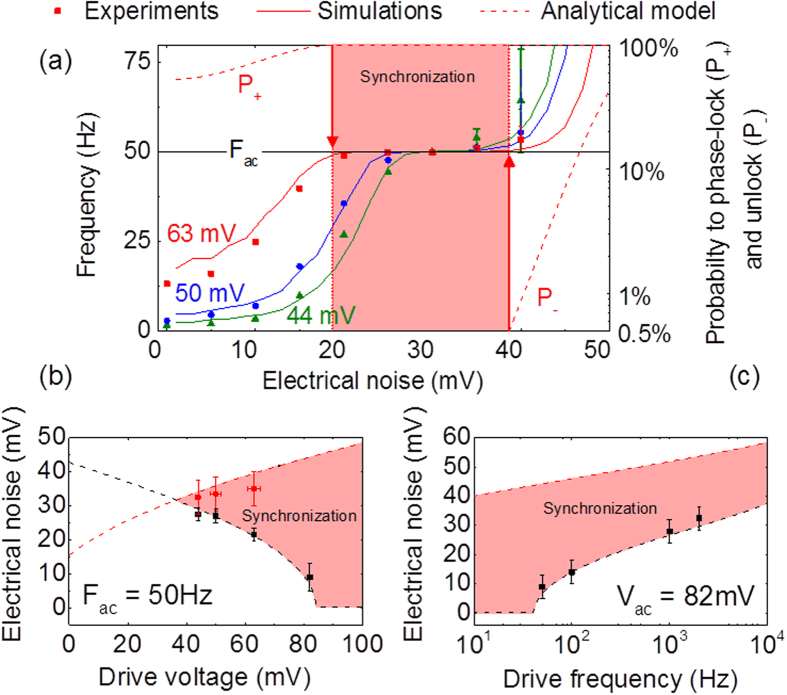
Modelling the phase locking of superparamagnetic tunnel junctions to an external periodic drive in the presence of electrical noise. Simulations and analytical calculations are done with the same set of parameters: V_c_ = 235 mV and ΔE/k_B_T = 22.5. (**a**) A square periodic voltage of frequency F_ac_ = 50 Hz and a white Gaussian electrical noise are applied to a magnetic tunnel junction. Three amplitudes are studied: V_ac_ = 44 mV (green), V_ac_ = 50 mV (blue) and V_ac_ = 63 mV (red). Left axis: frequency of the oscillator versus the standard deviation of the noise, both experimental results (circles, squares and triangles) and numerical results (solid lines) are represented. Right axis: analytical values of probabilities P_+_ and P_−_ to switch during half a period T_ac_/2 versus noise (dash lines). Vertical dot lines represent the noise levels for which P_+_ = 99.5% and P_−_ = 0.5% for a 63 mV amplitude. The horizontal black solid line represents the drive frequency F_ac_. (**b**,**c**) Lower noise bound (black) and higher noise bound (red) of the synchronization plateau versus the drive voltage (**b**) and versus the drive frequency (**c**). Both analytical values (dash lines) and experimental results (circles and squares) are presented. In the red zones the oscillator is synchronized with the excitation.

Phase-locking to an external drive controlled by electrical noise has been experimentally demonstrated in a few non-linear systems such as Schmitt triggers^19^,^21^ or lasers^22^ but never in a nanoscale system as achieved here. In order to assess the potential of superparamagnetic tunnel nanojunctions for applications, we now propose a model that accurately describes their noise-mediated synchronization to weak periodic signals. The thermally activated escape rate of a single domain magnetization, modulated by spin transfer torque^23^,^24^, has a simple expression^25^,^26^,^27^:


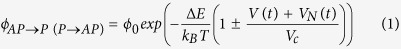


where *ϕ*_0_ is the attempt frequency, Δ*E* the energy barrier between the two stable states, *T* the temperature and *V*_*c*_ the voltage threshold for deterministic switching^26^,^27^. In our case the driving force is the sum of the periodic voltage *V*(*t*) = ±*V*_*ac*_ and the electrical noise *V*_*N*_(*t*), which is assumed Gaussian with standard deviation *σ*_*Noise*_. In consequence there are two sources of noise in our system: electrical and thermal noises (T = 300 K). Using Eq. (1), we can numerically compute the junction’s mean frequency as a function of the electrical noise amplitude^28^ (see Methods). Figure 2a compares the experimental data (symbols) measured for different amplitudes of the periodic drive to the results of numerical simulations (solid lines). All simulations have been performed using a single set of fitting parameters (*Vc* = 235 mV and Δ*E*/*k*_*B*_*T* = 22.5), emphasizing the remarkable agreement with experimental results.

The analytical models that have been developed in the past to describe noise-induced phase locking^18^,^20^,^29^ focused on cases for which noise can be taken into account as a time-independent variable in the escape rates, such as temperature in Eq. (1). However in our case, the escape rates from the parallel and antiparallel states are time varying, random variables because they depend on the electrical noise *V*_*N*_(*t*). In order to go further, we develop an original and generic method to analytically determine the conditions for synchronization. Starting from Eq. (1), we calculate the probabilities *P*_+_ (*P*_−_) for the magnetization to switch from out-of-phase with the drive voltage to in-phase (from in-phase to out-of-phase) during half a period. The details of the derivation and the expressions for the phase-locking and phase-unlocking probabilities *P*_+_ and *P*_−_ are given in Methods. In the vicinity of the plateau, the mean frequency of the junction is described by *F* = *F*_*ac*_(2*P*_+_ + 2*P*_−_ − 1). Considering a 99% frequency locking requirement, the boundaries of the synchronization region are given by *P*_+_ >99.5% and *P*_−_ <0.5%, as shown by the red arrows in Fig. 2a for the case of a drive amplitude of 63 mV. Figure 2b,c show that our analytical model (dotted lines) quantitatively predicts the boundaries of the experimental synchronization zone (symbols) over the whole range of investigated parameters. As can be seen in Fig. 2b, the range of electrical noise for which phase-locking occurs increases with the drive amplitude. When the drive amplitude is too low (here below 37 mV), synchronization cannot be achieved. On the other hand, at large drive amplitudes (here above 85 mV), phase locking can be achieved through room temperature thermal noise alone, without the need to add up electrical noise. In addition, as shown in Fig. 2c, phase-locking is achievable for frequencies orders of magnitude higher than the natural mean frequency (0.1 Hz here). The fact that synchronization occurs for broad ranges of noise amplitude provides robustness to variability of the device. For instance, if a drive voltage V_ac_ = 63 mV with frequency F_ac_ = 50 Hz is applied to the junction, a 5% variability of its area would shrink the synchronization noise range from 21 mV to 16 mV. This leaves a significant synchronization plateau that can be exploited for applications. Nevertheless, this variability increases the minimal drive amplitude required to achieve synchronization from V_ac_ = 31 mV to V_ac_ = 39 mV.

### Estimation of the energy needed to synchronize superparamagnetic tunnel junctions

Having validated our analytical model, we can now predict the energy consumption of spintronic circuits leveraging the synchronization of superparamagnetic tunnel junctions for computing. In such circuits, a calculation is finished once steady synchronization patterns are formed within the assembly of oscillators after its perturbation by an external input signal^15^,^16^. Superparamagnetic tunnel junctions can phase lock fast, typically in a single period of the input signal^30^. To evaluate the energy needed for such operation, we focus on the most recent generation of magnetic tunnel junctions with perpendicularly magnetized layers. We consider junctions small enough (<30 nm) for their free layer to behave as a macrospin^2^ and to be described by Eq. (1). Using parameters (energy barrier, critical voltage and resistance) determined from experiments by Sato *et al.*^2^, we calculate the minimum energy *E*_*min*_ necessary to synchronize the junction with and without the help of electrical noise.

**Figure 3:**
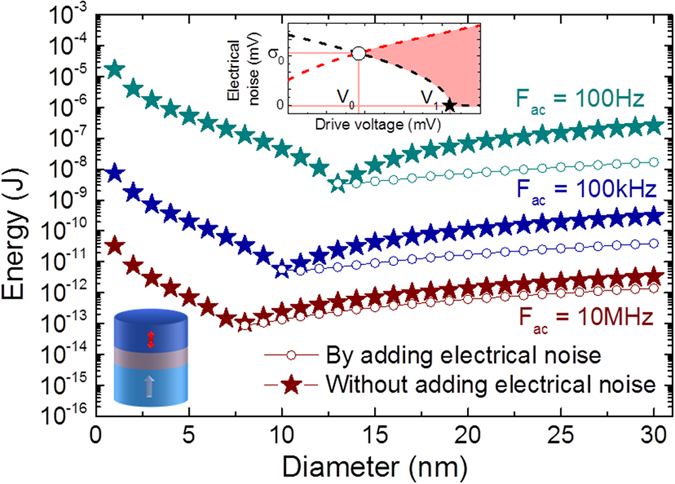
Energy required to phase-lock a perpendicularly magnetized superparamagnetic tunnel junction: predictions of the analytical model. Upper inset: schematic of Fig. 2b. The circle indicates the lowest drive voltage V_0_ for which synchronization can be achieved and the corresponding electrical noise level σ_0_. The star indicates the lowest drive voltage V_1_ for which synchronization can be achieved through thermal noise alone without addition of any electrical noise. Lower inset: schematic view of a perpendicularly magnetized tunnel junction. Main: Calculated minimum energy required to synchronize a perpendicularly magnetized superparamagnetic tunnel junction to a periodic voltage drive in one period, plotted versus the diameter of the junction, for different drive frequencies. Circles represent the case where electrical noise has been added while stars represent the case where only thermal noise is used.

Figure 3 shows the evolution of *E*_*min*_ as a function of junction diameter for different drive frequencies. When only thermal noise is used, 
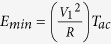
 where V_1_ is the minimum drive voltage required to phase-lock the junction in one drive period *T*_*ac*_ (see inset in Fig. 3 and Methods). When electrical noise is added, the minimum energy becomes 
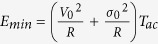
, where V_0_ is the minimum drive voltage required for phase-locking and σ_0_ the corresponding noise level (see inset in Fig. 3 and Methods). Interestingly, for each drive frequency, there is an optimal diameter *D*_*min*_ for which the energy needed to achieve phase-locking is minimal. Indeed the junction diameter determines its natural frequency: large diameters correspond to low frequencies because large magnetic volumes are more difficult to switch. Above *D*_*min*_, the drive frequency is larger than the junction’s mean frequency. To phase-lock, the junction has to be accelerated. In the absence of electrical noise, this can be done through an increase of the drive amplitude, which enhances the ability of the junction to synchronize (stars in Fig. 3).

Adding electrical noise lowers the drive amplitude required to synchronize and thus decreases the total amount of energy to provide (circles in Fig. 3). Below *D*_*min*_, the junction has to be slowed down in order to phase lock. As electrical noise always speeds up the oscillator by increasing the number of switches, this can only be achieved by increasing the drive amplitude. Our results indicate that carefully engineering the junctions’ dimensions can drastically decrease the energy required to achieve phase-locking, about 8 × 10^−14^ J for a drive frequency of 10 MHz (Fig. 3). By comparison, synchronizing a harmonic dc-driven spin-torque oscillator with a 10 GHz frequency^31^ to a drive current would require 100 times more energy (see Methods). CMOS implementations of oscillators for bio-inspired computing applications are also more costly in terms of energy, with a consumption above 7 × 10^−12^ J for integrate and fire neurons^32^. In addition they occupy a large area on chip, typically several hundreds of μm^2^.

Because of these issues of size and energy consumptions, bio-inspired computing systems leveraging the synchronization of coupled oscillators for computing have never been implemented in CMOS. Thanks to their small area and low energy consumption, arrays of phase-locked superparamagnetic tunnel junctions are a promising alternative for pattern recognition. We take the example of image classification, which generally requires one oscillator per pixel^15^,^16^ to evaluate the energy consumption of magnetic processor leveraging the synchronization of superparamagnetic tunnel junctions. Using the figures determined above, we predict that a superparamagnetic spintronic circuit can classify 1Mpixel images while consuming less than 0.1 μJ. A challenge for designing such computing circuits will be the generation and control of both the drive signal and the electrical noise. Towards this goal, only two levels of noise are sufficient to induce synchronized or non-synchronized states. One possible low power path would be to use the electrical noise naturally present in the circuits, such as, for instance, the noise generated by assemblies of stochastic devices. Our study thus opens the path to ultra-low power stochastic computation harnessing superparamagnetism.

## Methods

### Sample

The samples are in-plane magnetized magnetic tunnel junctions. They were fabricated by sputtering, with the stack: substrate (SiO_2_)/Buffer layer 35 nm/IrMn 7 nm/CoFe 2.5 nm/Ru 0.85 nm/CoFeB 2.4 nm/MgO-barrier 1.0 nm/CoFeB 1.7 nm/capping layer 14 nm. The whole stack was annealed before microfabrication at 300 °C under a magnetic field of 1 Tesla for 1 hour. Patterning was then performed by e-beam lithography, resulting in nanopillars with elliptic 60 × 120 nm^2^ cross-sections.

### Experiments

The electrical noise applied to the junction is white Gaussian noise with a bandwidth *F*_*N*_ = 40*MHz*. Measurements are performed under an in-plane applied field H_0_ of 59 Oe in order to compensate the residual stray field produced by the reference layer (synthetic antiferromagnet), and thus equilibrate dwell times in the P and AP states in the absence of applied voltage.

### Data analysis

In order to determine the resistance of the junction as a function of time, we record the current flowing through the junction with an oscilloscope. As the driven voltage oscillates between two values (+*V*_*ac*_ and −*V*_*ac*_) and the magnetic tunnel junction switches between two resistance states (R_AP_ = 640 Ω and R_P_ = 390 Ω), the current flowing through the junction can take four values. At high level of electrical noise, the determination of the resistance state of the junction becomes more difficult, as can be seen from the increasing width of error bars in Fig. 2a.

### Numerical simulations

We assume that the free layer of the superparamagnetic tunnel junction can be considered as a single domain magnetization element and follows the Neel-Brown model^25^ in which the escape rates of this process are described by Arrhenius equations and can be controlled through the handle of spin transfer torque^26^,^27^


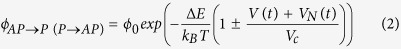


In this study *ϕ*_0_ = 10^9^ *s*^−1^ is the effective attempt frequency^27^, Δ*E* is the energy barrier between the two stable states, *k*_*B*_ is the Boltzmann constant, *T* is the temperature, *U*(*t*) = *V*(*t*) + *V*_*N*_(*t*) is the applied voltage and *V*_*c*_ is the threshold voltage for deterministic switching^26^,^27^.

For numerical simulations we compute at each time step the probability for the magnetization to switch during the time interval *dt* knowing the initial state:


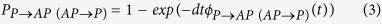


A pseudo-random number is then generated to decide whether the switch occurs or not. The parameters of the model: *F*_*ac*_, *V*_*ac*_ (frequency and amplitude of the driving square voltage) and *F*_*N*_ (bandwidth of the electrical Gaussian noise) have values identical to the experimental protocol. The chosen time step corresponds to the smallest time scale of the experimental noise generator *dt* = 1/*F*_*N*_. The mean frequency is computed as the mean number of oscillations of the junction per second.

Matching numerical predictions with experimental results for the evolution of the frequency of the magnetic tunnel junction versus the level of noise (Fig. 2(a) left axis) allows us to extract the two free parameters of the model: the ratio Δ*E*/*T* *=* 22.5 and the critical voltage *V*_*c*_ = 235 *mV*.

### Analytical model

The specificity of external noise is that it introduces a supplementary level of randomness as compared to the internal noise provided by temperature: the escape rates depend on the electrical noise *N*(*t*) and are therefore random variables themselves. Therefore, the probability for the magnetization to switch during half a period *T*_*ac*_/2 of the drive can only defined as an average over the possible values of N:


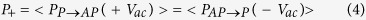



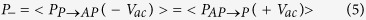


where 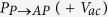 is the probability to switch from P to AP during *T*_*ac*_/2 when the excitation voltage is +*V*_*ac*_.

Therefore, *P*_+_ is the probability to switch from out of phase to in-phase during *T*_*ac*_/2 while *P*_−_ is the probability to switch from in-phase to out of phase during *T*_*ac*_/2.


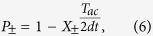


with


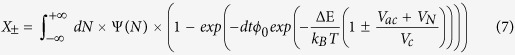


and Ψ(*N*) is a Gaussian distribution over N.

When the level of noise is sub-optimal, synchronization is limited by the junction’s ability to phase-lock fast enough when the excitation voltage reverses. Therefore the mean frequency of the junction is *F* = *F*_*ac*_(2*P*_+_ − 1). On the other hand, when the noise level is supra-optimal, synchronization is limited by the junction’s tendency to jump out of phase with the excitation voltage. Therefore *F* = *F*_*ac*_(1 + 2*P*_−_). On the whole, near the plateau, the mean frequency of the junction is *F* = *F*_*ac*_(2*P*_+_ + 2*P*_−_ − 1). In consequence, *P*_+_ > 99.5% and *P*_−_ < 0.5% means that the junction is frequency-locked with less than 1% error.

V_0_ is computed as the minimum voltage drive V_ac_ for which there is an electrical noise level σ_0_ that satisfies P_+_ > 99.5% and P < 0.5%. V_1_ is the minimum voltage drive V_ac_ for which P_+_ > 99.5% and P_−_ < 0.5% is satisfied at zero electrical noise.

### Energy consumption predictions

We consider a perpendicularly magnetized tunnel junction and make the assumption that the free layer is a single magnetization element (*D* < 30 *nm*). The thickness of the free layer in the junction is fixed, so that the energy barrier between the two magnetization states scales with the square of the diameter D: 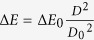. We also consider that the resistance × area product *RA* of the junction is constant, so that the resistance of the junction can be written as: 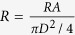. We use numerical parameters from Sato *et al.*^2^: Δ*E*_0_ = 90 *k*_*B*_*T*, *D*_0_ = 30 *nm*, *V*_*c*_ = 0.71 *V* and *RA* = 10 Ω.*μm*^2^.

For the energy consumption of a deterministic spin-torque oscillator we considered the same model with a resistance × area product *RA* = 3 Ω.*μm*^2^ and a diameter of 24 nm (which corresponds to the traditionally required energy barrier of Δ*E* = 60 *k*_*B*_*T*). The power consumption is dominated by the DC current required to induce high frequency oscillations of the magnetization, therefore 
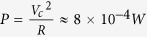
. Thus for a spin torque oscillator of 10 GHz frequency taking *T*_*sync*_ = 10 ns to reach synchronization^31^ the energy consumption is *E* = *P* × *T*_*sync*_ ≈ 8 × 10^−12^ *J*.

## Additional Information

**How to cite this article**: Mizrahi, A. *et al.* Controlling the phase locking of stochastic magnetic bits for ultra-low power computation. *Sci. Rep.* **6**, 30535; doi: 10.1038/srep30535 (2016).
